# Oviposition, Feeding Preferences and Distribution of *Delia* Species (Diptera: Anthomyiidae) in Eastern Canadian Onions

**DOI:** 10.3390/insects11110780

**Published:** 2020-11-11

**Authors:** Julia J. Mlynarek, Maggie MacDonald, Kathrin Sim, Kim Hiltz, Mary Ruth McDonald, Suzanne Blatt

**Affiliations:** 1 Montreal Insectarium, Montreal, QC H1X 2B2, Canada; 2 Harrow Research and Development Centre, Agriculture and Agri-Food Canada, Harrow, ON N0R 1G0, Canada; kathrin.sim@canada.ca; 3 Biological Sciences, University of Alberta, Edmonton, AB T6G 2E9, Canada; mbmacdon@ualberta.ca; 4 Kentville Research and Development Centre, Agriculture and Agri-Food Canada, Kentville, NS B4N 1J5, Canada; Kim.hiltz@canada.ca; 5 Department of Plant Agriculture, University of Guelph, Guelph, ON N1G 2W1, Canada; mrmcdona@uoguelph.ca

**Keywords:** root maggot, *Allium*, bioassay, community

## Abstract

**Simple Summary:**

*Delia* root maggot species are commonly found in onions. It is unclear which species affects onions most and how they are distributed among the major onion growing regions in Eastern Canada. Through oviposition and feeding preference bioassays, we determined that all species can similarly affect onion crops. We also determined that proportions of *Delia* species differ between the growing regions.

**Abstract:**

*Delia antiqua, Delia platura* and *Delia florilega* are three root maggot species commonly considered pests in Eastern Canadian onions. The onion maggot, *D. antiqua,* is considered the primary root maggot pest in onion but it remains unclear whether the other two species are also causing damage. In order to develop updated management strategies for root maggot, we tested adult oviposition and feeding preference by *Delia* larvae on four growth stages of onion using bioassays and we determined the *Delia* species composition across the four major onion growing regions in eastern Canada. *Delia* species oviposit readily on onion at the 5–7 true leaf growth stage but damage on onions is not statistically different between *Delia* species in our zero-inflated models. The four eastern Canadian onion growing regions have different proportions of *Delia* species. Southern Ontario and Quebec were the only two regions where *Delia antiqua* was collected. The highest average numbers of *Delia* spp. were caught in Quebec and Nova Scotia. Our study shows that timing is important in implementation of management strategies for root maggot in Eastern Canadian onions.

## 1. Introduction

Canadian field vegetable and field crops can be host to several damaging root maggot species in the genus *Delia* Robideau-Desvoidy (Diptera: Anthomyiidae) [1]. The two most studied pests are specialist species: *Delia radicum* (Linnaeus), the cabbage maggot, and *Delia antiqua* (Meigen), the onion maggot. *Delia radicum* feeds on members of the Brassiceae [2,3] while *D. antiqua* feeds on various *Allium* species. Other species within the genus, e.g., *Delia platura* (Meigen), the seedcorn maggot, and *Delia florilega* (Zetterstedt), the bean seed maggot, are considered generalists. *Delia platura* can develop on over 40 host plants in multiple families [4,5]. *Delia florilega* is polyphagous with the ability to feed and develop on legumes, garden crops and cereals [4,6].

Three *Delia* species are commonly associated with onion crops: *D. antiqua, D. platura* and *D. florilega* [1]. Larvae of *Delia* spp. damage plants by feeding on the germinating seeds, seedlings and, if conditions are right, can even mine the stems, rendering the plants unmarketable. Losses of untreated onion plants attributed to *D. antiqua* feeding can average 24–40% in Canada, depending upon variety [1]. Although *D. antiqua* is considered the primary pest of onions, studies have shown that *D. platura* and *D. florilega* may be causing a larger proportion of damage to onion bulbs than previously thought [7,8,9,10]. Certain studies, based on field observations, even suggest that *D. platura* and *D. florilega* may be primary invaders in onion [6,11].

Current management strategies targeting root maggots in eastern Canadian onion crops include chemical, cultural and biological controls, but are applied non-discriminately to all species of *Delia* maggots. Insecticides (usually cyromazine or chlorpyrifos) are commonly applied at seeding to control the first generation of root maggots in the spring; however, *D. antiqua* has been showing signs of resistance to chlorpyrifos [10,12]. This insecticide will no longer be available in Canada after 2020. Insecticide seed treatments are now available, but are applied prior to seeding, and also may affect all species of *Delia* whether they attack onions or not. Cultural controls such as appropriate crop rotation and timed planting potentially control root maggot populations [13], however crop rotation is not a feasible option in many of the intensive onion production regions of Canada. Biological controls measures such as entomopathogenic nematodes and fungi [14,15,16] may reduce root maggot damage. These strategies are used singly or in combination to reduce *Delia* pest pressure [13].

Even with multiple Integrated Pest Management (IPM) tools available to control root maggots, the probability of success is affected by the lack of knowledge about the species that are causing damage to the crops. The main objectives of our research were to use bioassays to (a) elucidate female preference to lay eggs at particular plant growth stages (hereafter oviposition), (b) determine feeding preference to identify which species actively damages onion and at what growth stage and (c) determine the diversity and distribution of *Delia* species in eastern Canadian onion growing regions (Nova Scotia, Quebec and Ontario). The combination of these will hopefully provide insight into management strategies for root maggots in onion.

## 2. Materials and Methods

### 2.1. Study Species

*Delia antiqua* and *D. platura* puparia were obtained from London Research and Development Centre (London, ON, Canada) and Montmorency College (Laval, QC, Quebec), respectively. *Delia florilega* was not included into the bioassays because we could not obtain or establish a laboratory colony for that species. *Delia* species were reared for one generation on an artificial diet as described in Ishikawa et al. [17] and, following Silver et al. [18], maintained in a growth chamber at 22 °C with 70% humidity and a photoperiod of 18:6, Light:Dark, at the Kentville Research and Development Centre (Agriculture and Agri-Food Canada).

Onion seed of *Allium cepa* cv. (LaSalle) was obtained from Stokes Seed, Thorold, Ontario, and sowed in plastic pots (4 cm diameter) with a standard greenhouse potting mix, watered as needed and maintained in the same growth chamber with the *Delia* colonies. When plants reached one of 4 different plant growth stages: (1) 5 days post seeding, (2) radical/flag leaf stage, (3) second true leaf stage (TLS) and (4) 5–7th true leaf stage (TLS), they were used in bioassays. The plant stages 2 through 4 lasted on average four days each.

### 2.2. Bioassays

#### 2.2.1. Oviposition Preference

For each replicate, one mating pair (approximately 48–96 h old) was introduced into a BugDorm-1 (BioQuip Products, Rancho Dominguez, CA, USA) with one onion plant for 48 h with 20 replicates for each growth stage and species combination. We chose 48–96 h old mating couples because that length of time allowed us to be certain that the females were mated and that they have not started laying yet. The plant was then destructively examined under a microscope for the number and placement of eggs (Supplementary Table S1).

#### 2.2.2. Feeding Preference

Onion plants were inoculated with zero, one or three *D. antiqua* or *D. platura* larvae, which had hatched 24–36 h prior to the assay, at the specific plant growth stages, with 20 replicates for each density and root maggot species. Larvae remained in pots until pupae or adult flies were recovered. Once the plants reached the bulb stage, they were assessed for damage (i.e., marketability; Supplementary Table S2). The damage rating was assessed on a scale from 0 to 5 based on an altered Dosdall’s scale [19] and grouped based on rating. Samples with ratings between 0 and 1 were deemed marketable, and ratings from 2 to 5 were deemed unmarketable. A control with no larval inoculation was also done, but these were not included in the analyses because no damage was observed in these controls.

#### 2.2.3. Statistical Analyses

The data for the oviposition bioassay was over-dispersed (variance = 57.16 > mean = 3.61) with an excess of zeros (76.3% zero counts) so we fitted a Zero-Inflated Generalized Linear Model with Negative Binomial distribution (ZINB) with plant growth stage and *Delia* species as explanatory variables and number of eggs laid as the response variable. It was the best-fit model compared with a Zero-Inflated Generalized Linear Model with Poisson distribution (ZIP) using Akaike Criterion (AIC) (ZIP AIC score 387.49 and ZINB AIC score 326.21).

Similarly, for the damage bioassays, the data were over-dispersed (variance = 4.75 > mean = 1.89) with an excess of zeros (46.9% zero counts). We fitted a ZIP Model with plant growth, larval density and *Delia* species as explanatory variables and mean damage rating as the response. It was the best-fit model when compared with a ZINB using AIC (ZIP AIC score 1031.18 vs. ZINB AIC score 1033.18).

All statistics were done with the pscl package [20,21] in RStudio [22].

### 2.3. Distribution

#### 2.3.1. Study Sites and Sampling

**Figure 1:**
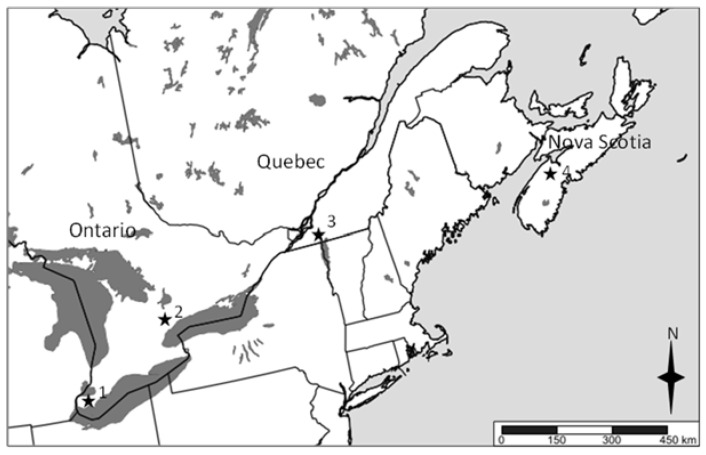
Main onion growing regions in Canada. Regions denoted by stars: 1—Southwestern Ontario, 2—Southern Ontario, 3—Quebec, 4—Nova Scotia.

The study was conducted in four regions (Figure 1) with six sites in Nova Scotia (45.0° N, 64.5° W; average precipitation 47.01), five sites in Southwestern Quebec (45.4° N, 73.2° W; average precipitation 82.85 mm), six sites at the Muck Research Station, University of Guelph in the Holland marsh, Ontario (44.1° N, 79.5° W; average precipitation 99.32) and five sites in the Southwestern region of Chatham-Kent County, Ontario (42.3° N, 82.5° W; average precipitation 11.67 mm). At each site, four dry-touch blue sticky cards (Solida, QC, USA), measuring 9.5 × 12.5 cm, were placed facing into the field on the edge of the crop a meter from the ground to collect adult *Delia* species flying through the field. We used blue sticky cards because that is the color of sticky card used by Canadian growers to monitor for onion maggot. These sticky cards were changed twice a weekly from planting (late May) through to harvest (late July) in 2017 (Supplementary Table S3). We also pulled wilted onion plants and collected soil around those onions to verify for maggots. However, no maggots were found.

Once the sticky cards were collected, they were returned to the laboratory. *Delia* species from the sticky traps were identified using the key provided by Savage et al. [6]. A subset of the sticky cards was sent to Bishop’s University for verification of species identity. A few specimens were removed from traps, mounted and deposited at the Canadian National collection as vouchers. Only males were identified because females of *D. platura* and *D. florilega* cannot be reliably distinguished morphologically, especially from sticky cards. Identification of female *Delia* relies on bristles on legs and other easily distorted or characters that can be broken.

#### 2.3.2. Statistical Analyses

For each region, the data were pooled over the entire season since there was little difference in diversity of *Delia* within each region. Spatial autocorrelation was tested using the Mantel test and a Permutational Multivariate Analysis of Variance (PermANOVA) based on Bray–Curtis distance matrix was used to determine significant differences among *Delia* species assemblages by region using the function Adonis. All statistics were run with the vegan R package [23] in RStudio [22].

## 3. Results

### 3.1. Bioassays

#### 3.1.1. Oviposition Preference

Eggs were deposited around the base of the plant or in the crevices of the plant and in some cases, both. This was consistent for all the bioassays. Only the Radical/flag plant growth stage (F = 3.29; *p* < 0.01) showed a significant difference with respect to ovipositional preference in the zero-inflation portion of the models (Table 1). It must also be noted that *Delia platura* did not oviposit on onions at any plant stage during the tested 48 h exposure.

#### 3.1.2. Feeding Preference

**Figure 2:**
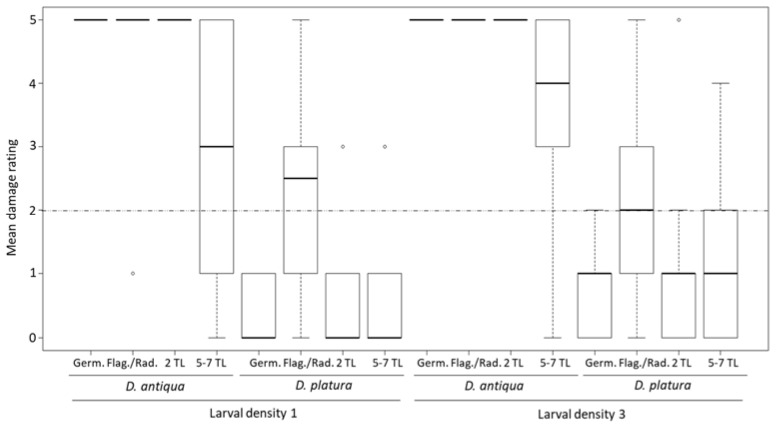
Feeding damage assessment across plant growth stages, and *Delia* species when inoculated with 1 or 3 *Delia* larvae in growth chamber experiments. Dotted line at 2 denotes the limit of marketability (below is marketable, above is unmarketable).

The significantly different factors in the Poisson count model portion are species (F = −1.26; *p* < 0.01) and 5–7 TL growth stage (F = −0.31; *p* < 0.01). A single *D. antiqua* caused onion to be non-marketable (mean damage rating of 3) when fed upon at the 5–7 TL stage. With 3 larvae, the damage rating increased to 4 (Figure 2). *Delia platura* caused some damage to onion when feeding on the radical/flag plant growth stage with a damage rating of 2. Feeding by *D. platura* on any other plant growth stage caused only minimal damage. There are no significant factors in the zero-inflation portion of the model (Table 2).

### 3.2. Distribution of Delia

**Figure 3:**
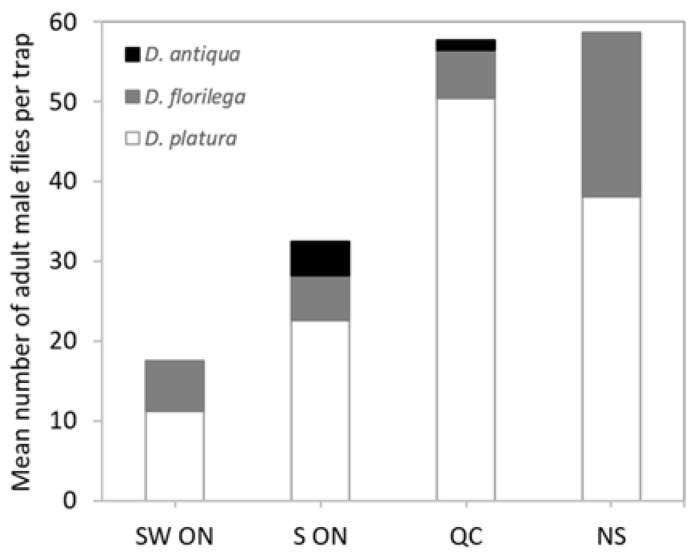
Mean number (for the collecting period) of adult male *Delia* species collected from blue sticky cards in four major onion growing regions of Canada.

Nova Scotia and Quebec had the highest number of adult *Delia* flies followed by Southern Ontario and Southwestern Ontario (Figure 3), but only Quebec and Southern Ontario had *D. antiqua*. *Delia platura* was the most common species in all regions. These differences in composition were statistically significant (PerMANOVA Adonis R^2^ = 0.50, *p* < 0.05). The Mantel test showed that spatial distance between the regions is not auto correlated with *Delia* diversity (r = 0.47; *p* = 0.001). *Delia* species composition is more similar within regions then between regions (Figure 3).

## 4. Discussion

This study shows some interesting results about the relative oviposition preference and damage to onions seedlings and bulbs caused by *Delia* root feeding maggots in eastern Canada. Both *Delia* spp. were found to be capable of causing economic damage to onions in laboratory bioassays, even when only a single larva was present, highlighting the risk root maggot species present.

*Delia antiqua* favor older onion plants for oviposition. This is in contrast with past studies and beliefs when it was widely thought that most oviposition occurs when onion plants are seedlings and most vulnerable [1] and foliar insecticide sprays of diazinon or chlorpyrifos to protect bunching onions are applied to seedlings in the spring. The variation in oviposition preference suggests management strategies should focus on monitoring for *Delia* adult population size when the onions are planted, followed by insecticide treatments when the onions are a little older.

Our results are consistent with those of Harris and Miller [24], who found that onion flies were most attracted to upright cylinders 2–12 mm in diameter, and oviposited seven times more on 15 cm high cylinders as compared to 2 cm. Our results are also consistent with Mowry [25] as oviposition was low on young onion seedlings. When given the choice of a seedling or a sprouted onion bulb, female *D. antiqua* deposited 60–200 times more eggs on the sprouted bulb than the seedling [25]. Ovipositing females choose the host plant and they must be able to recognize the suitability of the host plant for the development of their progeny. Dindonis and Miller [26] found decomposing onion seedlings and bulbs to elicit a greater host finding response by female *D. antiqua* than healthy plants. Healthy bulbing plants caught significantly fewer *D. antiqua* females than their decomposing counterparts, with healthy seedlings eliciting a response comparable to that of the decomposing seedlings. Preference for the decomposing onion may further be advantageous for larval survival due to easier larval penetration of onion bulbs and efficient larval development [26]. Monitoring and removing non-healthy onions will help reduce *Delia* spp. pressure by reducing oviposition.

*Delia platura* is a generalist and our results show that they are not significantly different from *D. antiqua* in their likelihood of feeding on onion. However, we noted that the adult *D. platura* do not oviposit eggs on onions, and, based on our observations, the larvae do not survive when they have to feed on onions. This is consistent with work on this species in other cropping systems: females oviposit into fields with decaying organic matter and the larva are attracted to germinating seedlings [27]. These results counter, in part, observations where *D. platura* is a serious pest of onion in California [10] and in the UK [11]. These findings are supported by experiments demonstrating that *D. platura* prefer decomposing onions [26], which could explain the ubiquitous nature of *D. platura* in the Canadian onion fields surveyed. *Delia platura* could be feeding on damaged and decomposing onions as well as other plants within the onion fields. As *D. platura* appears to be a lower risk pest, growers in Eastern Canada should focus on managing *D. antiqua.* This is contrary to other studies, and general knowledge, where *D. platura* is reported as a serious pest in onion in other regions [10,11]. The lack of *D. platura* impact on onion by populations from Nova Scotia and Quebec suggests there may be population genetic differences within the species or different environmental conditions to become an economic pest in some regions, e.g., California and the UK, but not others.

The community composition of *Delia* differs among the Eastern Canadian growing regions, and some regions are at a low risk of infestation by *D. antiqua*. The distribution survey found the main onion growing regions of Eastern Canadian to have different proportions of the three common *Delia* species. Southern Ontario had the highest proportion of *D. antiqua* followed by Quebec, while Nova Scotia and Southwestern Ontario did not have any *D. antiqua*. *Delia platura* was common on sticky traps in all regions but Nova Scotia and Quebec had higher proportions of that species when compared with Ontario. Differences in presence of *D. antiqua* are potentially due to field management, especially crop rotation [13], and the evolution of resistance [12]. *Delia antiqua* was collected only in Southern Ontario and Quebec, two regions where fields are intensely cultivated for onions year after year, and have been for at least eight decades, leading to similar population variability as suggested by Lamb and Boivin [28]. In Southern Ontario, growers generally alternate between onions and carrots primarily, and sometimes celery or another root vegetable is added to the rotation (Cranmer personal communication Ontario Ministry of Agriculture, Food and Rural Affairs (OMAFRA)). The rotations are similar in Quebec, but radishes and leafy greens may be added to their rotations (Van Dyk, personal communication OMAFRA). In Nova Scotia, onion crops are rotated intensely using a 7-year rotation from an onion crop to several non-allium crops, while in Southwestern Ontario, onion fields are in rotation frequently, but tend to space fields at large distances (at least 10 km) from one another from one year to another (Mlynarek personal observation Agriculture and Agr-Food Canada (AAFC)). Martinson et al. [29] concluded that long-range directed movement has little influence on success of colonization, but we believe in the case of Southwestern Ontario and Nova Scotia, the distance may just be too great between fields and years for the *Delia antiqua* to locate fields of onions. These types of host–non-host, large distance rotations change the communities of *Delia* spp. in the field where a specialist like *Delia antiqua* will not be present but generalists like *D. platura* and *D. florilega* are more abundant.

There are two main caveats of our exploration of the diversity and distribution of *Delia* species. We only measured the presence of males of each species of *D. platura* and *D. florilega* as females are more difficult, or impossible to confidently differentiate using morphological characters. Additionally, identification of *Delia* females relies on the number and position of bristles on the legs and thorax, which can be lost or damaged on sticky cards, leading to misidentifications [6]. The second caveat also has to do with our study solely focusing on assessing populations and diversity using sticky cards traps. We must reiterate at this point that we removed wilted looking plants and shifted soil around those plants to check for maggots, but did not find any. Sticky cards are the standard trapping method used by growers to scout *Delia* populations, and we employed them for our surveys, but they may not be the best trapping method. Broatch and Vernon [30] showed that pan traps collect more intact specimens than sticky cards. *Delia antiqua* is more attracted to yellow, rather than blue traps, but the use of yellow sticky cards in this study would only increase the numbers of *D. antiqua* and decrease the numbers of other *Delia* species trapped. Additionally, we cannot be sure that sticky trap catches accurately reflect larval populations in bulbs, or just reflect diversity of insects flying through the environment.

Further research to develop management strategies focused on *Delia* is needed for Eastern Canadian onion crops in line with the ones proposed by Vernon et al. [31]. *Delia antiqua* has developed resistance to some insecticides [12]. There is a need to reduce insecticide use, and to identify new modes of action to manage this pest. Some of the new seed treatments are used at very low rates per hectare and do not target the third generation of onion flies, so may reduce the risk of insecticide resistance. An integrated pest management approach is desirable, with combinations of chemical, biological and cultural methods, and crop rotations [13] where possible. Intercropping [32] can be effective for small or organic production systems. Delayed planting [33] can be effective for short-season onions or bunching onions but is not always practical for bulb onion production.

## 5. Conclusions

Through these findings, we suggest a three-fold approach to managing *Delia* spp.: (1) monitoring for *Delia* spp. should focus early in the planting season to determine if management of the crop is necessary before the vulnerable growth stage (5–7 TL), (2) monitoring and pulling non-healthy young onion plants to discourage *Delia* spp. from ovipositing and populations growing and (3) managing the field with chemical or biocontrol should focus when the plants are near a 5 TL stage if the *Delia* spp. populations are high. In order to prevent damage in onions by *Delia* spp. root maggots, biological control methods [11,14,15] should be researched.

## Figures and Tables

**Table 1:** Results of Zero-Inflated Generalized Linear Model with Negative Binomial distribution (ZINB) testing for oviposition preference between onion plant growth stage, *Delia* larval density and *Delia* species for damage to the onion plants.

Count Model Coefficients			
Factors	Coefficient Estimate	Std Error	*p*
Intercept	2.74	0.14	<0.01
Species	−0.69	0.61	0.25
Growth (5–7 TL)	0.03	0.18	0.88
Growth (germ)	−6.96 × 10^-10^	NA	NA
Growth (rad/flag)	−0.49	0.43	0.26
Log (Theta)	1.49	0.31	<0.01
**Zero-inflated coefficients**			
Intercept	−1.10	0.52	0.03
Species	22.84	4347.31	0.99
Growth (5–7 TL)	−18.79	4347.31	0.99
Growth (germ)	21.53	6095.67	0.99
Growth (rad/flag)	3.29	0.90	<0.01

**Table 2:** Results of Zero-Inflated Generalized Linear Model with Poisson distribution (ZIP) testing for feeding preference differences between onion plant growth stage, *Delia* larval density and *Delia* species for damage to the onion plants.

Count Model Coefficients			
Factors	Coefficient Estimate	Std Error	*p*
Intercept	1.49	0.10	<0.01
Species	−1.26	0.09	<0.01
Growth (5–7 TL)	−0.31	0.10	<0.01
Growth (germ)	−0.01	0.10	0.91
Growth (rad/flag)	0.15	0.09	0.09
Ldensity	0.03	0.03	0.42
**Zero-inflated coefficients**			
Intercept	20.64	69.72	0.77
Species	10.50	65.18	0.87
Growth (5–7 TL)	−0.42	1.01	0.68
Growth (germ)	0.55	0.85	0.52
Growth (rad/flag)	−10.28	57.18	0.86
Ldensity	−31.35	95.44	0.74

## Supplementary Materials

The following are available online at https://www.mdpi.com/2075-4450/11/11/780/s1, Table S1: Raw data for the *Delia* oviposition bioassay, Table S2: *Delia* feeding preference bioassay, Table S3: Raw data *Delia* distribution.
